# Laparoscopic‐assisted myomectomy: Surgery center versus outpatient hospital

**DOI:** 10.1111/jog.14197

**Published:** 2020-01-29

**Authors:** Natalya Danilyants, Mamta M. Mamik, Paul MacKoul, Louise Q. van der Does, Leah Haworth

**Affiliations:** ^1^ The Center for Innovative GYN Care Rockville Maryland USA; ^2^ Albert Einstein College of Medicine New York New York USA

**Keywords:** ambulatory surgery center, leiomyoma, laparoscopic, ligation, myomectomy

## Abstract

**Aim:**

To compare the safety protocols and operative outcomes of women undergoing laparoscopic‐assisted myomectomy (LAM) by the same surgeons at a freestanding ambulatory surgery center (ASC) versus a hospital outpatient setting.

**Methods:**

Retrospective chart review of all women ≥18 years old with symptomatic leiomyoma, who underwent LAM with uterine artery occlusion or ligation for blood loss control, at a freestanding ASC between 2013 and 2017, and an outpatient hospital setting between 2011 and 2013, both serving the metropolitan Washington, DC area. The procedures were performed by two minimally invasive gynecologic surgical specialists from a single practice. The safety protocols of each setting were reviewed to identify similarities and differences.

**Results:**

A total of 816 LAM cases were analyzed (ASC = 588, hospital = 228). The rate of complications was comparable across settings, as was the average myoma weight (ASC = 396.2 g; hospital = 461.5 g; *P* = 0.064). Operative time was significantly shorter at the ASC: 68 min (95% CI 66–70) versus 80 min at hospital (95% CI 76–84), *P* < 0.0001. Ambulatory surgery center and hospital protocols differed in limits of preoperative hemoglobin (minimum 9.0 g/dL, 7.5 g/dL respectively), lower nurse/patient ratio in PACU, and were similar in intraoperative surgical safety standards.

**Conclusion:**

Laparoscopic‐assisted myomectomy can be performed safely and effectively by skilled surgeons at a freestanding ASC, even in patients with morbid obesity or large leiomyoma.

## Introduction

Advances in technology and more emphasis on laparoscopic techniques in residency have resulted in a shift in gynecological surgery from the inpatient to the outpatient setting.^1^ This changing landscape of gynecologic surgery promotes further migration to ambulatory surgery centers (ASCs), freestanding outpatient facilities that allow for same‐day surgeries and patient discharge. Ambulatory surgery centers have demonstrated advantages of greater efficiency, patient convenience and lower costs than the hospital‐based outpatient surgery model.^2^, ^3^, ^4^, ^5^, ^6^ However, more research is needed to establish the safety and feasibility of performing advanced gynecologic procedures outside the hospital setting.

Uterine myomas are the most common gynecologic tumors in the United States, with a prevalence of 40% and up to 70% in women aged 35 years and 70 years, respectively.^7^ With improvement in laparoscopic techniques, minimally invasive myomectomy has gained wide acceptance as an alternative to abdominal myomectomy and hysterectomy in treating symptomatic leiomyomata for women desiring uterine or fertility preservation.^8^

Although past research has established the safety of same‐day discharge after minimally invasive myomectomy,^9^ there is limited data on performing this procedure at a freestanding ASC.^10^, ^11^ The high risk of intraoperative hemorrhage during myomectomy is one of the main concerns of performing this procedure at an ASC, where blood transfusion capabilities and subspecialty support are not typically present.

Numerous medical and surgical techniques have been developed to maintain hemostasis during nonhysteroscopic myomectomy.^12^ Pharmacological agents such as intravaginal prostaglandins and oxytocin infusion are often used to reduce blood loss. However, their effects may not be completely controlled and may have negative results. Vasopressin, for example, has a short half‐life of 10–20 min, and its use has led to rare but serious consequences such as bradycardia, cardiovascular collapse and even death.^13^, ^14^

Reversible uterine artery occlusion and permanent uterine artery ligation are two nonpharmacologic approaches to controlling blood loss during myomectomy that have been shown to lower estimated blood loss (EBL), transfusion rates, length of stay and myoma recurrence rates, without significant impact on fertility.^15^ The objective of our study is to compare safety protocols and operative outcomes of laparoscopic‐assisted myomectomy (LAM) performed at a freestanding ASC versus a hospital outpatient setting (hospital), using the blood loss control techniques of reversible uterine artery occlusion and permanent uterine artery ligation.

## Methods

We compared all consecutive LAM cases performed at an ASC from October 2013 to October 2017 to all consecutive LAM cases performed at a community hospital by the same private practice gynecology group from January 1, 2011 to December 31, 2013 in a retrospective review. The gynecology group consisted of two high‐volume laparoscopic surgical specialists, who performed all the procedures in the hospital and ASC cohorts.

The ASC in this study is a freestanding outpatient surgical center which opened on October 1, 2013, and serves both local patients from the greater metropolitan Washington, DC area, as well as patients who travel long‐distance from outside regions. The ASC is certified by Medicare, and accredited by the Accreditation Association for Ambulatory Health Care (AAAHC), an organization which has been granted its status as an accrediting body by the Centers for Medicare and Medicaid Services. All ASCs in the United States are expected to meet the set of standards set forth by the AAAHC, and there are currently over 7000 accredited ASCs.

The hospital is accredited by the Joint Commission on Accreditation of Healthcare Organizations, which has a similar role as AAAHC in determining eligibility for Medicare reimbursement and setting standards of safety for their respective healthcare settings. The safety standards and protocols for both the hospital and ASC were reviewed in April 2019.

The study received a waiver of informed consent for retrospective data collection from the respective hospital and ASC settings; Holy Cross Hospital Institutional Review Board (IRB), Reference number: 2011‐01 and IntegReview IRB, an independent review board service, Reference number: CIGC‐001. An IRB approval was not required for comparison of institutional safety protocols as these protocols do not involve any patient identifiers or interventions.

Study subjects were women ≥18 years old, nonpregnant, with symptomatic leiomyoma. The type of myomectomy performed in all cases was LAM, a laparoscopic‐assisted myomectomy with temporary uterine artery occlusion or uterine artery ligation, as described below. Following the opening of the ASC in October 2013, the location of surgery was dictated by the patient's insurance. There were no preoperative criteria (uterine size, body mass index [BMI], comorbid conditions, etc.) that would preclude the performance of LAM at the ASC.

Patient characteristics analyzed included age, race, weight, BMI and prior medical/surgical history. We used the Elixhauser Comorbidity Index, a set of 30 comorbidity indicators used to predict hospital resource use and in‐hospital mortality, to identify and record comorbid conditions that have been shown to have a potential impact on clinical outcomes.^16^

Clinical outcomes included EBL, perioperative times (skin‐to‐skin and anesthesia), myoma weight, pathology and intraoperative and postoperative complications. Blood loss estimations were conducted by the anesthesiologist by measuring the volume in the suction canister and the estimated absorption of any laparotomy sponge used, where each sponge absorbs about 100 mL.

We identified intraoperative complications from the surgeons' operative notes, defined as any deviation from the ideal intraoperative course occurring between skin incision and skin closure.^17^ Postoperative complication data were collected from the ASC and hospital records, including follow‐up phone calls and clinic appointments. Postoperative problems that required a visit to the emergency department, hospital readmission, or re‐operation were recorded as postoperative complications.

### LAM surgical technique

Laparoscopic‐assisted myomectomy, a technique first described by Nezhat in 1994, is a minimally invasive hybrid approach that combines the advantages of laparoscopy and laparotomy while minimizing the risks and limitations of both.^18^ LAM begins laparoscopically to allow adequate visualization of the anatomy, and enables blood loss control methods including the application of a tourniquet around the uterine isthmus for temporary uterine artery occlusion, and retroperitoneal dissection to ligate the uterine arteries at the origin. The subsequent minilaparotomy incision allows for thorough exploration and direct palpation of myomas for more complete removal, compared to a laparoscopic only approach, which may miss the smaller myoma due to lack of haptic feedback. Kalogiannidis *et al*. compared LAM to abdominal myomectomy, and found shorter operative time, decreased blood loss, shorter hospitalization and faster recovery in the LAM group.^19^ Prapas *et al*. compared LAM to laparoscopy, and found shorter operative time, removal of larger myoma, easier and faster uterine repair and a shorter uterine incision.^20^ Our previous study comparing LAM to abdominal and laparoscopic myomectomy found similar results as the aforementioned studies, but also included a robotic‐assisted laparoscopic myomectomy group in the study, which showed a greater rate of intraoperative complications, longer operative time and smaller number and weight of myoma removed than LAM. We also found LAM removed a significantly greater percentage of myoma with a submucosal component than the laparoscopic approaches, which is important where fertility is a concern.^21^

The blood loss control methods used by the surgeons in this study of reversible artery occlusion or permanent artery ligation are determined based on desired fertility, uterine size, number of myomas and complexity of the case. Temporary occlusion is generally performed when fertility is desired. If fertility is desired and a tourniquet cannot be placed, uterine artery ligation is performed, as this method has not been shown to impact future fertility.^15^ A detailed description of the procedure is described below.

Following direct entry technique, a 5 mm supraumbilical skin incision is made, the abdomen is elevated and the laparoscope trocar/cannula system is introduced through the incision. Once abdominal entry is confirmed, the trocar is removed and the 5 mm laparoscope is reintroduced into the abdomen. After initial abdominopelvic survey, an additional 5 mm suprapubic port is placed under direct visualization. A ZUMI uterine manipulator (CooperSurgical, Inc.) is used in all cases. To accomplish uterine artery occlusion, a latex‐based rubber catheter (or nonlatex in patients with latex allergies) is used as a tourniquet, and is placed laparoscopically through the broad ligaments and around the uterine isthmus. The suprapubic incision is then extended either in a transverse or vertical direction to 3–5 cm, depending on the exposure needed, followed by placement of a small or medium wound retractor. This allows the wound diameter to stretch to 6–8 cm, similar to a smaller open incision. The tourniquet is then tied down securely around the isthmus of the uterus causing temporary occlusion of the bilateral uterine arteries. This technique limits blood flow and decreases pulse pressure to the uterus. In cases where fertility is not desired, and in cases where the tourniquet does not provide adequate hemostasis or cannot be applied due to complex anatomy, uterine artery ligation is performed. To ligate the uterine artery, retroperitoneal dissection is performed to seal and divide the uterine artery at its origin from the anterior branch of the internal iliac. This is performed using a Harmonic scalpel (Ethicon, Inc.). The remainder of the procedure is then performed through the mini‐laparotomy. Leiomyoma are localized visually or with palpation, and small uterine incisions are made. Leiomyoma are then removed either intact or by manual segmentation above the fascia at the level of the skin, minimizing the risk of scattering leiomyomatous fragments below the fascia and into the abdominopelvic cavity. The uterus can be externalized if needed, and all uterine defects, including posterior, are hand‐sewn and closed in layers using the standard abdominal myomectomy closure technique. The tourniquet is released upon closure of the uterine defects. Any areas of inadequate hemostasis are immediately visualized and oversewn. Final hemostasis is confirmed on laparoscopic survey at the end of the procedure. Fascia and skin incisions are closed using absorbable sutures.

In both settings, the American College of Obstetricians and Gynecologists recommendations for enhanced recovery after surgery pathways were followed to facilitate improved pain control and recovery.^22^ Patients fasted for 6 h prior to surgery. No bowel prep was used. On arrival, patients were given acetaminophen 1 g and an opioid by mouth. A scopolamine patch was placed for patients with a history of severe nausea/vomiting. At the end of the procedure, the incisions were infiltrated with subcutaneous bupivacaine 0.25%. Postoperatively, patients received IV ketorolac with IV narcotics as needed. Prior to discharge home, they were given an oral nonsteroidal anti‐inflammatory drug (NSAID). Patients were deemed stable for discharge after adequate mobilization, tolerance of PO and pain control. The patients were then discharged home with NSAIDS and oral narcotics.

Patients who required transfer from the ASC to the hospital were provided transportation at the expense of the ASC via a local medical transport company to one of three hospitals located within 10 miles of the ASC. The hospital emergency department was contacted in advance to alert of the incoming patient.

Phone calls were made to all hospital and ASC patients on postoperative day 1. In cases where the patient could not be reached, follow‐up phone calls were attempted through postoperative day 7. All local patients were seen 2 weeks after surgery. ASC travel patients were seen 2–3 days postoperatively for follow‐up before returning home.

### Statistical analysis

The data were checked for potential outliers and aberrant measurements prior to inferential analyses. Patient demographic and clinical characteristics were measured on nominal or ordinal scales, and compared between surgical settings using Pearson's chi‐squared tests. Variables measured on an interval scale were compared across surgical settings using Student's *t*‐test.

Operative outcomes were compared between surgical settings after adjusting for patient demographic (e.g., race, age) and case complexity factors (e.g., previous abdominal surgery, comorbidities, average aggregate fibroid weight). We used median regression to model operative times and EBL because of concerns about non‐normality of the dependent variable. Length of stay and number of ports were compared using negative binomial regression. Logistic regression was used to model intraoperative, postoperative complications and conversions. All statistical analyses were conducted with spss 21 (IBM Inc.). All statistical tests were two‐tailed at the *P* < 0.05 level.

## Results

A total of 588 patients underwent LAM at the ASC and 228 patients at the hospital. While age, weight and BMI were comparable between the groups, there was a higher percentage of African‐American patients at the hospital than at the ASC (Table 1).

**Table 1:** Patient characteristics

	ASC	Hospital	*P*‐value
	*n* = 588	*n* = 228	
Age group, *n* (%)			0.7093
<30	51 (8.6)	28 (12.2)	
30–39	349 (59.4)	128 (55.9)	
40–49	181 (30.8)	69 (30.1%)	
50–59	6 (1.0)	4 (1.8)	
Race, *n* (%)			<0.0001
White	115 (19.6%)	28 (12.3%)	
Black	301 (51.2%)	175 (76.8%)	
Other	77 (13.1%)	24 (10.5%)	
Unknown	95 (16.2%)	1 (0.4%)	
Number of previous myomectomies, *n* (%)			0.2911
None	510 (86.7%)	188 (82.5%)	
1	71 (12.1%)	36 (15.8%)	
2 or more	7 (1.2%)	4 (1.8%)	
Number of previous abdominal surgeries, *n* (%)			0.3217
None	355 (60.4%)	129 (56.6%)	
1	170 (28.9%)	77 (33.8%)	
2	39 (6.6%)	17 (7.5%)	
2 or more	24 (4.1%)	5 (2.2%)	
Number of comorbidities, *n* (%)			0.4219
None	235 (40.0%)	90 (39.5%)	
1	200 (34.0%)	76 (33.3%)	
2	93 (15.8%)	35 (15.4%)	
3 or more	60 (10.2%)	27 (11.8%)	

	Mean ± SD	Mean ± SD	*P*‐value
Weight (kg)	76.8 (18.7)	76.1 (18.6)	0.6383
BMI (kg/m^2^)	28.3 (6.4)	27.9 (6.6)	0.3929
Fibroid weight (g)	396.2 (438.8)	461.5 (466.6)	0.0643

ASC, ambulatory surgery center; BMI, body mass index; g, grams; kg, kilograms; kg/m^2^, kilogram per meter squared; *n*, number; SD, standard deviation; yrs, years.

**Figure 1:**
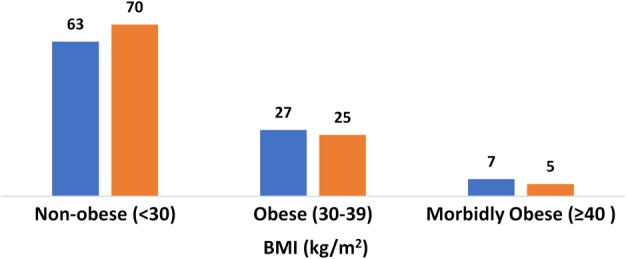
BMI by setting (%). (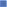) ASC, (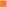) Hospital

**Figure 2:**
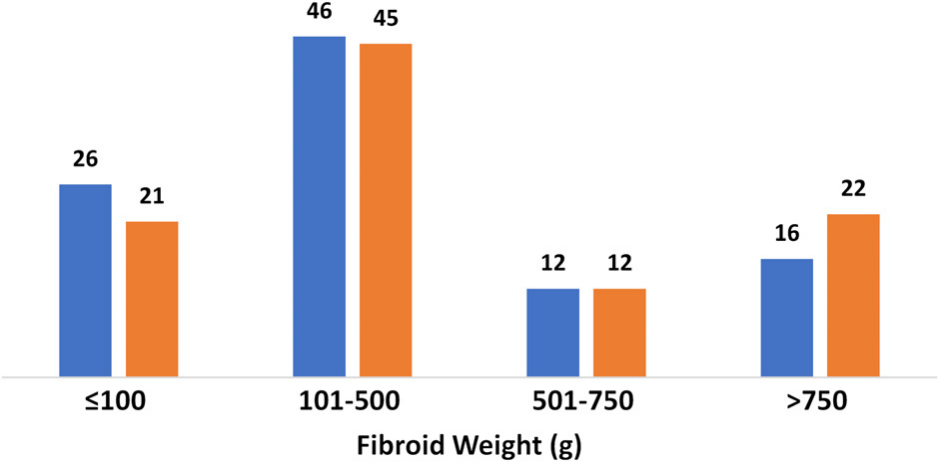
Aggregate fibroid weight by setting (%). (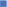) ASC, (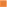) Hospital

There was no significant difference in case complexity factors between settings, including BMI, number of previous abdomino‐pelvic procedures or comorbidities (Table 1 and Fig. 1). There was also no statistically significant difference in average fibroid weight between settings (Table 1). The distribution of the aggregate fibroid weight removed per patient was evenly distributed across settings (Fig. 2), with a maximum fibroid weight per patient of 4426 g at the ASC compared to 3046 g at the hospital. The number of fibroids removed was similar in both settings, with a range of 1–82 at the ASC, and 1–103 at the hospital. Similarly, the range of the size of the largest fibroid was 1–26 cm at the ASC and 1–20 cm at the hospital.

The same‐day discharge rate from the ASC was 98%, compared to 70% at the hospital. Five ASC patients (0.9%) required transfer to the hospital, four for blood transfusions and one for postoperative asthmatic reaction with low oxygen saturation. All five patients recovered without further complication. In both the adjusted and unadjusted analyses, the blood transfusion rate was significantly lower at the ASC: 2% (95% CI .8–3.2) versus 6.5% at the hospital (95% CI 3.0–10.1), *P* < 0.0247 (Tables 2 and 3).

**Table 2:** Operative outcomes (unadjusted analysis)

	Setting				
	ASC		Hospital		*P*‐value
	*n*	Mean (SD)	*n*	Mean (SD)	
Estimated blood loss (mL)	582	199.8 (227.2)	227	265.0 (370.5)	0.3218
Length of stay (days)	588	0.0 (0.0, 0.0)	228	0.5 (1.0)	0.9753
Total surgery time (min)	579	69.9 (26.3)	226	81.3 (29.0)	<0.0001
Number of ports	573	2.2 (0.5)	223	2.4 (0.9)	0.0058

		*n* (%)		*n* (%)	
Intra‐op complications, *n* (%)	588	18 (3.1%)	228	11 (4.8%)	0.2222
Post‐op complications, *n* (%)	588	25 (4.3%)	228	16 (7.0%)	0.1046
Blood transfusion, *n* (%)	588	10 (1.7%)	228	19 (8.3%)	<0.0001
Conversion, *n* (%)	588	0 (0.0%)	228	2 (0.9%)	0.0231

**Table 3:** Operative outcomes (adjusted analysis)†

	Setting		
	ASC	Hospital	*P*‐value
	*n* = 588	*n* = 228	
Estimated blood loss (mL) ‐ adj. medians (95% CI)	153.3 (139.0–167.6)	147.2 (122.2–172.2)	0.6948
Operative time (min) ‐ adj. medians (95% CI)	67.8 (65.5–70.1)	80.0 (76.0–84.0)	<0.0001
Number of ports ‐ adj. counts (95% CI)	2.2 (2.1–2.4)	2.4 (2.2–2.6)	0.2912
Intra‐op complications, % (95% CI)	3.4 (1.8–5.0)	4.9 (1.7–8.1)	0.4430
Post‐op complications, % (95% CI)	4.9 (3.1–6.7)	5.9 (2.6–9.1)	0.6288
Blood transfusions, % (95% CI)	2.0 (0.8–3.2)	6.5 (3.0–10.1)	0.0247

† Adjusted for age, race, number of previous surgeries, body mass index, number of comorbidities, number of additional procedures, fibroid weight, number of previous myomectomies and uterine artery ligation/occlusion.

Adj, adjusted; ASC, ambulatory surgery center; CI, confidence interval; intra‐op, intraoperative; min, minutes; mL, milliliter; post‐op, postoperative.

The rate of intraoperative complications between settings was not significantly different, with 3.4% (95% CI 1.8–5.0) at the ASC compared to 4.9% (95% CI 1.7–8.1), *P* = 0.4430, at the hospital. There was also no statistically significant difference in EBL between settings. Nor were significant differences noted in postoperative complication rates between settings: 4.9% (95% CI 3.1–6.7) at the ASC versus 5.9% (95% CI 2.6–9.1), *P* = 0.6288, at the hospital (Tables 2 and 3). There were two conversions to standard laparotomy in the hospital setting and none at the ASC. There were no conversions to hysterectomy in either setting. The most common complications in each setting are listed in Table 4.

**Table 4:** Most common complications

Complications†, *n* (%)	Setting	
	ASC	Hospital
	*n* = 588	*n* = 228
Intraoperative		
Intraoperative hemorrhage (EBL > 1000 mL)	9 (1.5)	6 (2.6)
Blood transfusion	N/A	5 (2.2)
Bowel injury	5 (0.8)	2 (0.9)
Conversion to laparotomy	0 (0.0)	2 (0.9)
Uterine artery laceration	2 (0.3)	1 (0.4)
Bladder injury	1 (0.2)	0 (0.0)
Postoperative		
Blood transfusion	10 (1.7)	20 (8.8)
Fever	0 (0.0)	3 (1.3)
Intra‐abdominal bleeding	1 (0.2)	3 (0.3)
Ileus/small bowel obstruction	0 (0.0)	2 (0.9)
Abdominal wall hematoma	1 (0.2)	2 (0.9)
Emergency department for abdominal pain	6 (1.0)	1 (0.4)
Shortness of breath, chest pain	0 (0.0)	1 (0.4)
Pelvic fluid collection (drained)	0 (0.0)	1 (0.4)
Syncope	1 (0.2)	1 (0.4)
Incisional bleeding	2 (0.3)	1 (0.3)
Hypoxia	0 (0.0)	1 (0.4)
Nausea/vomiting	2 (0.3)	0 (0.0)
Pelvic abscess	1 (0.2)	0 (0.0)
Hemothorax	1 (0.2)	0 (0.0)

† Includes multiple intraoperative complications as dictated in operative notes, but counted as one event in statistical analysis.

The only meaningful difference in operative outcomes was the perioperative times. The average operative time was significantly shorter at the ASC: 68 min (95% CI 66–70) versus 80 min (95% CI 76–84), *P* < 0.0001 (Table 3) at the hospital. The difference in anesthesia times at the ASC (99 min 95% CI 97–101) compared to the hospital (113 min 95% CI 109–117), *P* < 0.0001 was also significant.

Surgical safety protocols were similar for both the hospital and ASC (Table 5). A noted difference at the ASC, however, is the lack of intraoperative blood transfusion capability, and the procedures implemented to account for this. Just prior to surgery in the ASC, blood counts are acquired with a handheld fast‐acting blood analyzer, with a minimum preoperative hemoglobin level of 9.0 g/dL. By contrast, the minimum required hemoglobin is 7.5 g/dL in the hospital, taken within 30 days of surgery.

**Table 5:** Safety protocols

Safety standards	Setting	
	ASC	Hospital
Preoperative	(1) Hemoglobin cut‐off of 9.0 g/dL. (2) I‐STAT used just prior to surgery on the day of surgery. (3) LAM cases are only scheduled as morning cases to allow for ample observation and recovery time.	(1) Blood count from within 30 days suffices. (2) Hemoglobin cut‐off of 7.5 g/dL. (3) No limitations in timing of scheduling LAM cases.
Intraoperative	(1) No blood transfusion capability. (2) Transfer to contracted hospitals if blood transfusion required. (3) Aseptic techniques, infection control and surgical sterility procedures are the same as the hospital setting.	(1) Blood transfusion available.
Postoperative	(1) 1:1 nurse/patient ratio (2) 23 h observation is available at ASC. (3) Thromboprophylaxis follows the same protocol as the hospital except high risk patients may go home with portable SCDs. (4) Travel patients required to stay in hotel near ASC and return for follow up 2–3 days postop. (5) Local patients return for follow‐up 2 weeks post‐op.	(1) >1:2 nurse/patient ratio. (2) Unlimited inpatient observation. (3) Patients with high risk for thrombosis must remain inpatient for SCD use. (4) Patients return for office follow‐up 2 weeks post‐op.

Another difference in safety protocols between settings is LAM cases are only scheduled in the morning at the ASC, in order to allow for ample observation and recovery time before same‐day discharge. Also, there is a 1:1 nurse/patient ratio in the ASC recovery room, compared to the hospital where the ratio is routinely higher. A 23 h observation is available at the ASC if needed.

Thromboprophylaxis practices are similar in each surgical setting, with thromboembolic disease (TED) hose and sequential compression devices (SCD) available for patients based on the level of risk (low, moderate, or high). However, patients at high risk who require SCDs after discharge from the ASC are sent home with portable, battery operated SCDs, whereas in the hospital these patients are admitted and monitored for adequate thromboprophylaxis until discharge.

Patients who travel long‐distance for surgery at the ASC are required to stay at a nearby hotel, and are seen at the ASC for follow‐up 2–3 days after surgery before traveling home.

## Discussion

The current study shows that skilled, high‐volume surgeons can safely perform LAM in a freestanding ambulatory surgical environment, where subspecialty back‐up and immediate blood transfusion capabilities do not exist. The low average EBL, low complication rate and lack of conversion to full laparotomy or hysterectomy across settings are evidence that this hybrid approach to myomectomy, combined with the blood loss control techniques of uterine artery occlusion and ligation, allow for the safe removal of large tumor loads.

The average operative and anesthesia times at the ASC were significantly shorter than the hospital. Shorter perioperative times were also reported in a retrospective study by Hair *et al*., which found significantly shorter surgery times, operating room times and postoperative times at freestanding ASCs compared to hospital‐based outpatient surgery centers across surgical specialties.^23^ These differences support the theory that ASCs are more efficient in their processes.

The difference in perioperative times between settings may also be attributed in part to improvements in surgical techniques over time, as the first 2 years of data in review were collected only in the hospital setting, and the ASC data was more recent, with only a 3 month overlap in data collection time from October to December 2013.

There was also a significantly lower blood transfusion rate at the ASC compared to the hospital. This is likely explained by the different criteria for preoperative blood transfusion between the two settings. In the hospital, the requirement for preoperative blood transfusion was set at a hemoglobin of less than 7.5 g/dL. Consequently, patients with a preoperative hemoglobin just above this limit were more likely to require a transfusion intraoperatively or postoperatively. However, for patients at the ASC, the preoperative transfusion criteria was less than 9.0 g/dL in order to limit the degree of preoperative anemia as blood is not available intraoperatively. We did not collect preoperative transfusion or postoperative hemoglobin levels, which we acknowledge is a study limitation.

The importance of surgeon experience also cannot be overlooked, with previous studies showing an association between high‐volume surgeons and lower surgical complications, as well as same‐day discharge.^24^ The two surgeons who performed all surgeries in this study are both experienced, high‐volume surgical specialists who are especially proficient in the reported minimally invasive techniques. But while past studies have emphasized the importance of proper patient selection to ensure safe outcomes at an ASC,^10^ our data show similar patient characteristics across the two settings, including comorbidities and BMI.

With rising rates of both obesity and ambulatory surgery in the United States over the past 20 years, there has been concern about increased surgical risks for obese patients in the ambulatory setting. While there is evidence that patients with a BMI less than 40 kg/m^2^ can safely undergo ambulatory surgery, and that there is increased risk of perioperative complications in the super morbidly obese (BMI > 50 kg/m^2^), there is limited data on the surgical outcomes for morbidly obese patients with BMI between 40 and 50 kg/m^2^.^25^ In our study, 7% of the patient population of our ASC was considered morbidly obese, compared to 5% in the hospital (Fig. 2), with no significant difference in complications or outcomes between settings.

The number of ASCs in the United States has grown exponentially since the opening of the first center in 1970, in response to the increased demand for an alternative to the inpatient hospital model. Advantages of ASC include shorter operative and facility times, less potential exposure to nosocomial infections and intensified quality control processes.^3^, ^4^, ^5^, ^6^ Surgeons also have greater autonomy in an ASC than in a hospital, enabling them to design customized surgical environments and hire specialized, highly efficient staff. Surgeons in an ASC are also more likely to be assigned a single operating room for all cases, resulting in fewer delays and quicker room turnover, and allowing the surgeon to perform a higher volume of cases in a shorter amount of time. In addition, hospitals are more likely to have emergency cases that compete with outpatient procedures for operating room time, interrupting patient flow and adding additional time and costs.^6^

Our study shows that advanced, minimally invasive surgical procedures such as LAM can be safely performed in a freestanding ambulatory surgical setting under certain recommended conditions. First, the primary or attending physician should be an experienced, high‐volume surgeon who is proficient in the planned surgical technique. Second, the patient should be hemodynamically stable, with a preoperative hemoglobin level ≥ 9 g/dL on the day of surgery. Third, surgical cases should be scheduled in the morning to allow for ample postoperative observation and recovery time before same‐day discharge. Ideally, the nurse‐patient ratio should be 1:1 in the postanesthesia care unit, to ensure effective postoperative recovery before discharge. Fourth, for patients at high risk of TED, portable battery operated SCDs to take home after same‐day discharge are recommended for thromboprophylaxis. Finally, in case of serious postoperative complications, established procedures should be in place for immediate transport to a hospital for additional care.

### Limitations

The retrospective nature of this study is limited by the availability and accuracy of the medical records. All hospital and ASC visits within 60 days of surgery were recorded, but patients may have been seen at a different facility or physician's office, resulting in a potential underreporting of postoperative complications. Also, as noted, the surgeons in this study are experienced, gynecologic laparoscopic specialists, whose outcomes may not be representative of the general gynecologic surgical community.

We also acknowledge the lack of cost data in this study. While the focus of our study was on the safety and feasibility of performing LAM at a freestanding ASC, future studies should include direct cost comparisons between settings. In an effort to push the healthcare system in the direction of greater sustainability, comparisons of surgical settings should also be framed in the context of value, examining the costs relative to outcomes and patient satisfaction, rather than analyzing individual metrics alone.^26^

The patient characteristics and surgical outcomes were comparable between the ASC and hospital study groups, supporting the idea that LAM can be safely performed by skilled surgeons in a freestanding ambulatory setting, without limitations in patient complexity. With the stated advantages of the ASC setting, this study contributes to the growing body of evidence that ASCs are both a viable and safe setting for advanced gynecologic procedures, and are key to meeting the growing demand for outpatient surgery.

## Disclosure

The authors declare they have no conflict of interest. Data abstractors were independent contractors with no vested interest in The Center for Innovative GYN Care. Dr Natalya Danilyants is a co‐owner of The Center for Innovative GYN Care, and while she contributed to the writing and editing of the manuscript, she did not play a role in the collection, analysis, or interpretation of the data. Dr. Louise van der Does is employed by The Center for Innovative GYN Care. At the time of writing and editing the manuscript, Dr Mamta M. Mamik was employed by The Center for Innovative GYN Care. Leah R. Haworth is an independent contractor.
